# David Alfred Alexander

**Published:** 1948

**Authors:** 


### Obituary

### DAVID ALFRED ALEXANDER, M.B., CH.B. (Victoria).

A NATIVE of Carnoustie, David Alfred Alexander was educated at Birkenhead and at Liverpool University : he won the Gee prize for Diseases of Children at Liverpool Royal Infirmary. He qualified in 1898 and next year settled in Bristol. After a short interval he bought the practice in Redfield where he has worked for over forty years. Though somewhat impatient of exaggeration and trivialities, he was well known for his meticulous care of the sick, regardless of status, paying two or even three visits a day to patients when he felt this necessary. He was universally respected and will be deeply mourned. Many can tell stories of his kindness and not a few of monetary help in need. He was an enthusiastic member of the Medico-Chirurgical Society and often contributed cases to clinical evenings. He published a number of papers in our *Journal*—" Abdominal Fibroma " (1908), " Cretinism and the Puerperium " (1909), " Raynaud's Disease " (1912)—and elsewhere ; " Portal Pyæmia " (*Lancet,* 1911), " Hyperpiesia " (*B.M.J.*, 1925) : a book of medical reminiscences is now in the hands of publishers. He had many other interests, especially in the work of St. Mary-le-Port Church, where he was for years a churchwarden, and a staunch defender of the right of the old church to restoration after the destruction in 1940. His end came suddenly at the age of seventy-five while he was cycling alone in the Quantocks he loved so well. He is survived by his widow and seven children : one son (G. L. A.) is in practice in New Zealand. To them and to his partner Dr. L. M. Houghton we tender our sincere sympathy.

